# Sleeve gastrectomy as a bridge to cardiac recovery - A retrospective comparative cohort study

**DOI:** 10.1016/j.jhlto.2025.100224

**Published:** 2025-02-07

**Authors:** Thomas Goubar, Samuel Kim, David Cistulli, Douglas Fenton-Lee, R. Louise Rushworth, Peter S. Macdonald, Anne M. Keogh

**Affiliations:** a St Vincent’s Hospital Sydney, 390 Victoria St, Darlinghurst, NSW 2010, Australia; b The University of Notre Dame, School of Medicine, Sydney, 160 Oxford St, Darlinghurst, NSW 2010, Australia; c Northern Beaches Hospital, 105 Frenchs Forest Rd W, Frenchs Forest, Sydney, NSW 2086, Australia; d Royal Prince Alfred Hospital, 50 Missenden Rd, Camperdown, Sydney, NSW 2050, Australia

**Keywords:** sleeve gastrectomy, heart failure with reduced ejection fraction (HFrEF), bariatric surgery, obesity management, cardiac transplant

## Abstract

**Background:**

Obesity in patients with heart failure with reduced ejection fraction (HFrEF) increases morbidity and may preclude them from accessing advanced heart failure therapies. Bariatric surgery, specifically sleeve gastrectomy (SG), may improve eligibility for cardiac transplant; however, its impact on heart failure outcomes is not well defined.

**Methods:**

We conducted a retrospective cohort study of patients with obesity (body mass index [BMI] ≥35 kg/m^2^) and (left ventricular ejection fraction [LVEF] ≤40%) who underwent SG at a tertiary heart transplant center. Outcomes were compared with controls matched for age, sex, LVEF, and BMI receiving standard care. We evaluated BMI, New York Heart Association (NYHA) functional class, medications, echocardiographic parameters, time to advanced heart failure therapies, and survival.

**Results:**

Twenty patients (median BMI 42.8 kg/m², LVEF 25%) underwent SG compared to 40 matched patients. Both groups demonstrated reductions in BMI; however, weight loss was significantly greater in the treatment group (−9.9 [95% CI −12.2, −7.6] vs. −2.7 [−4.3, −1.1] kg/m², p < 0.05). Despite this, improvements in LVEF (+16.6% [10.2, 23.0] vs. +0.1% [−4.4, 4.7], p < 0.05) along with NYHA class (−0.8 [95% CI: −1.3, −0.3] vs. +0.4 [0.1, 0.7], p < 0.05) were only observed in those receiving SG. Overall survival was significantly higher in the treatment group [HR: 0.2 (0.07, 0.62), p < 0.01], which had no deaths compared to 35% in the comparison group.

**Conclusion:**

In patients with HFrEF and obesity, SG is associated with significant improvements in cardiac function and survival compared to standard care, supporting its role as a safe and effective bridge to recovery or candidacy.

## Introduction

The intersection of obesity and heart failure, particularly heart failure with reduced ejection fraction (HFrEF), is an ongoing challenge in contemporary cardiology.^1^ Obesity is an independent risk factor for the development of heart failure, worsens the course of HFrEF and limits access to life-saving interventions like cardiac transplantation.^2^, ^3^ Recent evidence indicates that greater adiposity increases HFrEF related morbidity and mortality, contrasting earlier studies suggesting a protective effect of obesity on mortality in advanced heart failure.^4^, ^5^, ^6^, ^7^ The International Society of Heart Lung Transplantation eligibility guidelines for cardiac transplant recommend a body mass index (BMI) less than 35 kg/m^2^ due to higher perioperative complications and poorer outcomes with obesity.^8^, ^9^, ^10^ However, weight loss through conservative measures is difficult for patients with reduced exercise tolerance and heart failure symptoms.^11^

Bariatric surgery is a safe and effective obesity treatment which results in significant and durable weight loss.^12^, ^13^ Laparoscopic sleeve gastrectomy (SG) is preferred for its efficacy and favorable side effect profile compared to other bariatric procedures.^14^, ^15^ Bariatric surgery has been used to a limited extent in heart transplant centers as a bridge to transplant candidacy, in patients with left ventricular assist device (LVAD), with studies demonstrating the safety and efficacy of this approach.^16^, ^17^, ^18^, ^19^, ^20^, ^21^, ^22^ Fewer studies have assessed bariatric surgery in patients with advanced heart failure and comorbid obesity prior to bridging therapies, with early data suggesting successful bridging, however limited systematic studies have been performed.^23^, ^24^, ^25^, ^26^

The aim of the present study is to comprehensively evaluate the role of SG in the management of patients with obesity and HFrEF by comparing the mortality and morbidity, including a range of heart failure specific outcomes, between patients undergoing SG with a matched sample of patients with HFrEF receiving standard treatment.

## Materials and Methods

### Study design, setting and participants

A retrospective cohort study was conducted to evaluate the impact of SG on patients with obesity and HFrEF. Patients from a public tertiary referral heart transplant hospital in Australia with obesity (BMI ≥ 35 kg/m^2^) and HFrEF (LVEF ≤ 40%) who underwent SG between the years 2013 to 2022 were selected into the treatment group. All treatment group patients were assessed for SG suitability by a multidisciplinary team (MDT) through the Advanced Heart Failure Clinic. This team included heart failure cardiologists, cardiac anesthetists, and specialist bariatric surgeons, ensuring that only those deemed appropriate proceeded to surgery. The comparison group consisted of patients with obesity and HFrEF who received standard care at the same institution and were matched to the treatment group according to sex, age (± 5 years), BMI (± 5 kg/m^2^) and LVEF ((± 5%) in a 2:1 ratio. Of the 24 patients who received SG, three were excluded due to incomplete data, and one was excluded due to prior LVAD placement. Additionally, one patient developed severe aortic regurgitation during the study and, together with their matched controls, was excluded from echocardiographic analysis. The final cohort included 20 patients in the treatment group and 40 in the matched comparison group. Ethics approval was provided by St Vincent’s Hospital (2020/ETH01525) and the University of Notre Dame Australia Human Research Ethics Committee (021–037S).

### Outcomes

The primary outcomes were changes in BMI, LVEF, NYHA class and overall survival. Secondary outcomes included changes in cardiac medications and the following echocardiographic parameters: Left Ventricular End-Diastolic Diameter (LVEDD, mm); Left Ventricular End-Systolic Diameter (LVESD, mm); Left Atrial (LA) Diameter (mm); Interventricular Septal Thickness in Diastole (IVST, mm); Left Ventricular Posterior Wall Thickness in Diastole (LVPWd, mm); Right Ventricular Mid Diameter (RV mid diameter, mm); Fractional Shortening (FS, %); Left Ventricular Mass Index (LVMI, g/m²); and Relative Wall Thickness (RWT). Progression to advanced heart failure therapies, including successful listing for transplant, LVAD implantation and cardiac transplantation were also assessed.

All patients had at least two echocardiograms available for review prior to undergoing advanced heart failure therapies (LVAD implantation, Transplant). Echocardiograms pre- and post-surgery were included to assess changes following SG in the treatment group.

Heart failure medications were all agents used in heart failure management. Guideline-directed medical therapy (GDMT) comprised a count of any of the four pillars of heart failure management (0 to 4).^27^ Follow-up time and survival were identified through HealthENet, a state-wide clinical portal which shares summary level patient and clinical information, including prescription dispensing, and mortality. Patients were censored at the date of last contact with a healthcare provider.

### Statistical analysis

Data were collected using Microsoft Excel (Version 16.83; Microsoft Corporation, Redmond, WA, USA) and analysed using R Statistical Software (Version 4.3.1) in R Studio (Version 2023.9.1.494). Figures were produced using GraphPad Prism (Version 10.1.1). Baseline characteristics are presented as median (IQR) for continuous variables and counts (%) for categorical variables. Group comparisons were performed using Wilcoxon rank-sum test for continuous variables and Fischer’s exact test for categorical variables. Outcomes were assessed using a mixed-effects model, estimated via Restricted Maximum Likelihood (REML) and presented as mean [95% CI]. The model included fixed effects for treatment and time, with random intercepts for subjects to account for repeated measures. A compound symmetry covariance structure was assumed. Kaplan-Meier survival curves were generated to evaluate differences in overall survival, listing-free survival, LVAD-free survival, and transplant-free survival between groups, with the log-rank test used for comparisons. Censoring occurred at the event date or the last patient contact, whichever was earlier. P-values for outcome variables were adjusted utilizing a Bonferroni correction for multiple comparisons. A p-value of 0.05 was considered statistically significant.

## Results

### Baseline characteristics

Between September 2013 and June 2023, we identified a treatment group of 20 patients with HFrEF who underwent SG (80% male, n = 16), and a comparison group of 40 patients who received standard care (80% male, n = 32). Baseline characteristics and comorbidities were broadly comparable between groups (Table 1). Median age across both groups was 49.0 years (IQR 33.8–55.0). All patients had a baseline BMI > 35 kg/m², with a median BMI of 41.1 kg/m² (38.2–43.5). Median LVEF was 25.0% (15.0–30.0) and most patients in both groups were classified as NYHA class II (43.9%) or III (45.6%). The majority of patients had diabetes (61.7%), obstructive sleep apnea (58.3%) or atrial fibrillation (53.3%).

**Table 1:** Baseline Characteristics of Patients with HFrEF and Obesity Undergoing Sleeve Gastrectomy (Treatment) Versus Standard of Care (Comparison)

Demographics	Treatment (N = 20)	Comparison (N = 40)
Male sex — no. (%)	16 (80.0)	32 (80.0)
Age — yr	47.5 [30.5, 55.0]	49.0 [37.5, 55.0]
BMI (kg/m²)	42.8 [40.4, 44.1]	40.7 [38.0, 43.2]
Heart Failure Characteristics		
LVEF (%)*	25.0 [15.0, 32.5]	22.5 [15.0, 30.0]
NYHA class — no. (%)		
I	0 (0.0)	3 (7.5)
II	8 (40.0)	18 (45.0)
III	10 (50.0)	18 (45.0)
IV	2 (10.0)	1 ( 2.5)
Age at diagnosis — yr	35.0 [26.0, 50.2]	46.0 [34.8, 50.0]
Duration of heart failure — yr	3.0 [2.0, 5.0]	1.0 [0.0, 6.8]
DCM — no. (%)	19 (95.0)	38 (95.0)
Etiology — no. (%)		
Alcohol	0 (0.0)	2 ( 5.0)
Chemotherapy	0 (0.0)	1 ( 2.5)
Congenital	0 (0.0)	1 ( 2.5)
Familial	1 (5.0)	3 ( 7.5)
Idiopathic	8 (40.0)	21 (52.5)
Ischemic	8 (40.0)	8 (20.0)
Peripartum	0 (0.0)	1 (2.5)
Rheumatic	0 (0.0)	1 (2.5)
Viral	3 (15.0)	2 ( 5.0)
Advanced Heart Failure Therapies		
CRT — no. (%)	4 (20.0)	8 (20.0)
AICD — no. (%)	14 (70.0)	24 (60.0)
LVAD — no. (%)	0 (0.0)	0 (0.0)
Eligible not yet listed — no. (%)	7 (35.0)	24 (60.0)
Comorbidities		
Total comorbidities	4.0 [1.8, 6.2]	4.0 [2.0, 5.0]
Hypertension — no. (%)	9 (45.0)	19 (47.5)
Hyperlipidemia — no. (%)	12 (60.0)	17 (42.5)
Diabetes — no. (%)	12 (60.0)	25 (62.5)
IHD — no. (%)	7 (35.0)	10 (25.0)
CKD — no. (%)	8 (40.0)	20 (50.0)
OSA — no. (%)	12 (60.0)	23 (57.5)
GORD — no. (%)	5 (25.0)	14 (35.0)
AF — no. (%)	12 (60.0)	20 (50.0)
COPD — no. (%)	6 (30.0)	8 (20.0)
Pharmacotherapy		
Total medications	9.0 [7.0, 14.0]	10.5 [8.0, 12.0]
Heart failure medications	4.0 [3.0, 5.2]	4.0 [3.0, 5.0]
GDMT	3.0 [2.8, 3.0]	2.0 [2.0, 3.0]
Oral hypoglycaemic agents — no. (%)	13 (65.0)	16 (40.0)
GLP−1 agonist — no. (%)	0 (0.0)	5 (12.5)

* Echocardiographic measurements were based on a subset of control (n = 38) and treatment (n = 19) patients.

Continuous variables are presented as median [IQR], and categorical variables are presented as n (%). P-values for continuous variables were calculated using the Wilcoxon rank-sum test, and P-values for categorical variables were calculated using Fisher’s exact test. No calculated P-values reached significance.

The median age at diagnosis of heart failure was 43.0 years (30.8–50.0) and median duration of heart failure was 2.0 years (0.0–5.2). Most patients had a dilated phenotype (95%), with a median LVEDD of 67.0 mm (60.0–74.0). The most common etiology of heart failure was idiopathic (35%), and although a higher proportion in the treatment group had ischemic etiology (40%) compared to the comparison group (20%), this difference was not significant (p = ns) (Table 1). Patients were on a median number of 4.0 (3.0–5.0) heart failure agents, with the majority (56.7%) on three or more GDMT agents, and 30.0% on two GDMT agents. GLP-1 agonist use was recorded in 12.5% (n = 5) of the comparison group, all of which had T2DM, and no patients in the treatment group (p = ns). No patients had been listed for transplantation or received an LVAD at the time of first echocardiogram.

### Primary outcomes

**Figure 1:**
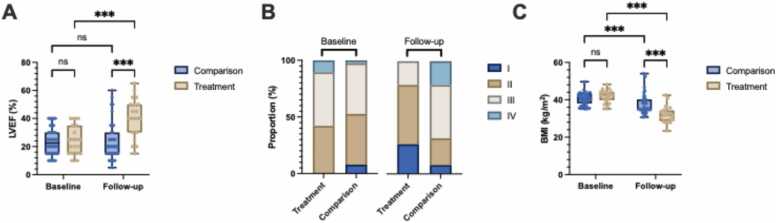
Primary Outcomes: Changes in (A) LVEF (%), (B) NYHA Functional Class and (C) BMI (kg/m^2^), Following Sleeve Gastrectomy (Treatment, n = 20) in Matched Patients with HFrEF and Obesity Compared to Standard Care (Comparison, n = 40). Sample size for LVEF: Treatment (n=19) and Comparison (n=38). Data are presented as box plots showing the median and the range from minimum to maximum values, or as stacked bar charts showing the proportion of patients in each group. P-values were calculated using a mixed-effects model with repeated measures (REML). Significant differences were identified using Bonferroni correction for multiple comparisons, with p-values as follows: p < 0.001 (***), p < 0.01 (**), **p** < 0.05 (*), and **p ≥ 0.05 (ns)**.

Follow-up duration and the interval between echocardiograms were similar between groups (Table 2). LVEF improved by 16.6% [95% CI: 10.2, 23.0] (p < 0.001) in the treatment group, but was unchanged (0.1% [−4.4, 4.7], p = ns) in the comparison group (between group difference: 16.5% [9.6, 23.3], p < 0.05) (Table 2;Figure 1A). Similarly, there was a mean reduction of −0.8 [−1.3, −0.3] (p < 0.001) in NYHA class in the treatment group, whilst NYHA increased by 0.4 [0.1, 0.7] (p < 0.05) in the comparison group (between group difference: −1.2 [−1.7, −0.6], p < 0.05) (Table 2;Figure 1B). Additionally, no deaths were recorded in the treatment group, compared to 14 (35%) in the comparison group (p < 0.05). There was a −9.9 kg/m² [−12.2, −7.6] (p < 0.001) reduction in BMI in the treatment group, and a smaller −2.7 kg/m² [−4.3, −1.1] (p < 0.001) reduction in the comparison group (between group difference: −7.2 kg/m² [−9.6, −4.7], p < 0.05) (Table 2;Figure 1C).

**Table 2:** Outcomes of Patients with HFrEF and Obesity Undergoing Sleeve Gastrectomy (Treatment) Versus Standard of Care (Comparison)

Clinical Outcomes	Treatment (N = 20)	Comparison (N = 40)	Adjusted p-value
Follow-up duration — yr (median [IQR])	5.0 [3.8, 7.2]	4.0 [1.0, 6.0]	ns
Δ BMI (kg/m²)	−9.9 [−12.2, −7.6]***	−2.7 [−4.3, −1.1]***	< 0.05
Δ NYHA class	−0.8 [−1.3, −0.3]***	0.4 [0.1, 0.7]*	< 0.05
Successfully listed for transplant — no. (%)	4 (20.0)	13 (32.5)	ns
Change in LVAD status — no. (%)	2 (10.0)	10 (25.0)	ns
Transplanted — no. (%)	3 (15.0)	12 (30.0)	ns
Died — no. (%)	0 ( 0.0)	14 (35.0)	< 0.05
Echocardiography*			
Interval between echos — months (median [IQR])	19.0 [12.5, 51.5]	13.0 [4.0, 35.8]	ns
Δ LVEF (%)	16.6 [10.2, 23.0]***	0.1 [−4.4, 4.7]	< 0.05
Δ FS (%)	7.2 [2.0, 12.5]**	−1.3 [−5.0, 2.4]	ns
Δ LVEDD (mm)	−4.6 [−8.4, −0.8]*	0.6 [−2.1, 3.3]	ns
Δ LVESD (mm)	−8.4 [−13.5, −3.2]***	1.5 [−2.2, 5.1]	< 0.05
Δ LVMI (g/m²)	−2.6 [−17.8, 12.6]	12.5 [1.7, 23.2]*	ns
Δ LA diameter (mm)	−3.5 [−6.9, −0.2]*	2.4 [0.1, 4.8]*	< 0.05
Δ IVST (mm)	−0.1 [−1.0, 0.8]	0.1 [−0.6, 0.7]	ns
Δ LVPWd (mm)	−0.3 [−1.3, 0.6]	0.3 [−0.4, 0.9]	ns
Δ RWT	0.01 [−0.02, 0.05]	0.01 [−0.02, 0.03]	ns
Δ RV mid diameter (mm)	−2.2 [−4.8, 0.4]	1.0 [−0.8, 2.8]	ns
Δ Aortic Root Diameter (mm)	1.1 [−0.8, 3.0]	1.3 [0.1, 2.7]*	ns
Medications			
Δ Total medications	−1.7 [−3.26, −0.03]*	0.7 [−0.5, 1.8]	ns
Δ Heart failure medications	−1.0 [−1.7, −0.2]**	0.2 [−0.4, 0.7]	ns
Δ GDMT	−0.1 [−0.4, 0.3]	0.2 [−0.1, 0.4]	ns
Δ Oral hypoglycaemic agents	−0.6 [−1.0, −0.2]**	0.2 [−0.1, 0.5]	< 0.05

* Echocardiographic measurements were based on a subset of control (n = 38) and treatment (n = 19) patients.

Continuous variables are presented as mean [95% CI], unless otherwise specified and categorical variables are presented as n (%). P-values for continuous variables were calculated using a mixed effects model, while P-values for categorical variables were calculated using Fisher’s exact test. Significant P-values for within group comparisons are presented as follows: p < 0.001 (***), p < 0.01 (**), p < 0.05 (*). A Bonferroni correction was applied to adjust for multiple comparisons.

No intraoperative complications were recorded, although three patients experienced the following post-operative complications within 7 days of the SG procedure: hematoma around the sleeve site necessitating a return to theater, minor surgical site bleeding and thrombocytopaenia.

### Secondary outcomes

**Figure 2:**
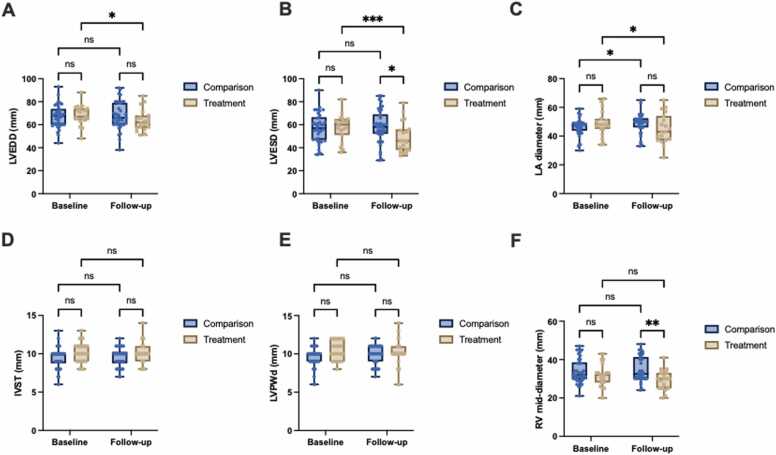
Changes in Cardiac Structure in Patients with HFrEF and Obesity Undergoing Sleeve Gastrectomy (Treatment) Versus Standard of Care (Comparison). Comparison of (A) Left Ventricular End-Diastolic Diameter (LVEDD, mm), (B) Left Ventricular End-Systolic Diameter (LVESD, mm), (C) Left Atrial (LA) Diameter (mm), (D) Interventricular Septal Thickness (IVST, mm), (E) Left Ventricular Posterior Wall Thickness in Diastole (LVPWd, mm) and (F) Right Ventricular (RV) mid-diameter (mm) at Baseline and Follow-up in Treatment (n = 19) Versus Comparison (n = 38). Data are presented as box plots showing the median and the range from minimum to maximum values. P-values were calculated using a mixed-effects model with repeated measures (REML). Significant differences were identified using Bonferroni correction for multiple comparisons, with p-values as follows: p < 0.001 (***), p < 0.01 (**), p < 0.05 (*), and p ≥ 0.05 (ns).

**Figure 3:**
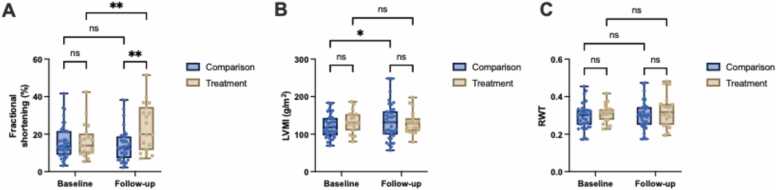
Changes in Derived Parameters of Cardiac Structure and Function in Patients with HFrEF and Obesity Undergoing Sleeve Gastrectomy (Treatment) Versus Standard of Care (Comparison). Comparison of (A) Fractional Shortening (FS, %), (B) Left Ventricular Mass Index (LVMI, g/m²), and (C) Relative Wall Thickness (RWT) at Baseline and Follow-up in Treatment (n = 19) Versus Comparison (n = 38). Data are presented as box plots showing the median and the range from minimum to maximum values. P-values were calculated using a mixed-effects model with repeated measures (REML). Significant differences were identified using Bonferroni correction for multiple comparisons, with p-values as follows: p < 0.001 (***), p < 0.01 (**), p < 0.05 (*), and p ≥ 0.05 (ns).

LV dilatation improved in the treatment group, with a mean reduction in LVEDD of −4.6mm [−8.4, −0.8] (p < 0.01), while there was no change (0.6 mm [−2.1, 3.3], p = ns) in the comparison group (between group difference: −5.2 mm [−9.2, −1.1], p = ns) (Table 2; Figure 2A). LVESD was reduced in the treatment group −8.4 mm [−13.5, −3.2] (p < 0.001) and unchanged in the comparison sample (1.5 mm [−2.2, 5.1], p = ns) with a between group difference of −9.8 mm [−15.4, −4.3] (p < 0.05) (Table 2; Figure 2B). There was an improvement in LA diameter (−3.5 mm [−6.9, −0.2], p < 0.05) in the treatment group, whilst this deteriorated by an average of 2.4 mm [0.1, 4.8] (p < 0.05) in the comparison group (between group difference: −6.0 mm [−9.5, −2.4], p < 0.05) (Table 2; Figure 2C). LV systolic function improved in the treatment group, with FS increasing by 7.2% [2.0, 12.5] (p < 0.01), while FS was unchanged (−1.3% [−5.0, 2.4], p = ns) in the comparison group (between group difference: 8.5% [3.0, 14.1], p = ns) (Table 2 & Figure 3A). In contrast, there was an increase in LVMI of 12.5 g/ m^2^ [1.7, 23.2] (p < 0.05) in the comparison group, which was not observed in the treatment group (−2.6 g/m^2^ [−17.8, 12.6], p = ns) with a between group difference of −15.1 g/m^2^ [−31.2, 1.1] (p = ns) (Table 2; Figure 3B).

**Figure 4:**
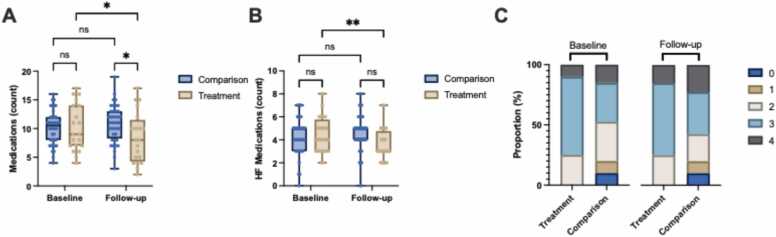
Changes in Medication Use in Patients with HFrEF and Obesity Undergoing Sleeve Gastrectomy (Treatment) Versus Standard of Care (Comparison). Comparison of (A) Total Medications (count), (B) Heart Failure (HF) Medications (count), and (C) Proportion of Patients Receiving Guideline-Directed Medical Therapy (GDMT) (Graded 0 to 4) at Baseline and Follow-up in Treatment (n = 20) Versus Comparison (n = 40). Data are presented as box plots showing the median and the range from minimum to maximum values, or as stacked bar charts showing the proportion of patients in each group. P-values were calculated using a mixed-effects model with repeated measures (REML). Significant differences were identified using Bonferroni correction for multiple comparisons, with p-values as follows: p < 0.001 (***), p < 0.01 (**), p < 0.05 (*), and p ≥ 0.05 (ns).

There was a mean reduction of −1.7 medications [−3.26, −0.03] (p < 0.05) in the treatment group, while medication use did not change significantly in the comparison group (0.7 medications [−0.5, 1.8], p = ns) with a between group difference of −2.3 medications [−4.0, −0.6] (p = ns) (Table 2; Figure 4A). Additionally, treatment patients had a mean reduction in oral hypoglycaemic agents of −0.6 medications [−1.0, −0.2] (p < 0.01), and remained unchanged (0.2 medications [−0.1, 0.5], p = ns) in the comparison group (between group difference: −0.8 medications [−1.2, −0.3], p < 0.05) (Table 2). Of the Comparison group patients receiving GLP-1 agonists (n=5), all experienced weight loss, with a median treatment duration of 17.5 months (IQR 13.7–42.1 months). Four of the five patients achieved a BMI below 35, with a mean BMI reduction of 5.47 kg/m² (SD ± 3.08, p = 0.017). Total heart failure medications diminished in the treatment group (−1.0 medications [−1.7, −0.2] (p < 0.01), while there was no change (0.2 medications [−0.4, 0.7] (p = ns) in the comparison group (between group difference: −1.1 medications [−1.9, −0.3], p = ns) (Table 2; Figure 4B). GDMT use remained stable in both groups (p = ns) (Table 2; Figure 4C).

### Bridge to candidacy

At follow-up four patients (20%) in the treatment group and 13 (32.5%) in the comparison had been successfully listed for transplant, with a median time to listing of 1.4 years (0.8–6.7) for treatment and 0.5 years (0.2–3.5) for comparison for those who were listed (p = ns). Of these, two patients (10%) in the treatment group and 10 (25%) in the comparison group went on to receive an LVAD, with a median time to LVAD of 4.3 years (0.5–8.1) for treatment group and 0.1 (0.0–1.1) for comparison group for those who received an LVAD (p = ns). Of patients listed, three patients (15%) in the treatment and 12 (30%) in the comparison went on to cardiac transplantation, with a median time to transplant of 1.2 years (0.7–1.9) for treatment and 1.3 years (0.4–3.9) for comparison for those who underwent transplantation (p = ns).

**Figure 5:**
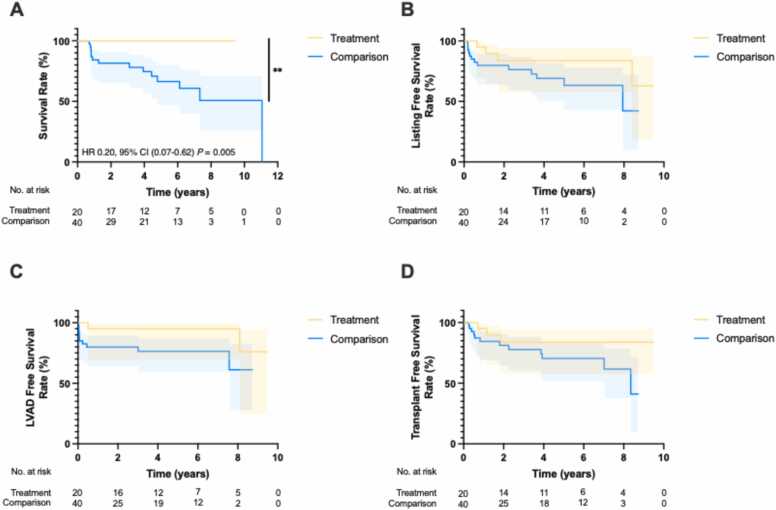
Kaplan-Meier Survival Curves Comparing Outcomes Between Patients with HFrEF and Obesity Undergoing Sleeve Gastrectomy (Treatment) Versus Standard of Care (Comparison). Kaplan-Meier curves depict (A) Overall survival, (B) Listing-free survival, (C) LVAD-free survival, and (D) Transplant-free survival in patients undergoing sleeve gastrectomy (Treatment, n=20) versus standard of care (Comparisonl, n=40). Shaded areas represent 95% confidence intervals. Survival rates were compared using the log-rank test, with p-values as follows: p < 0.001 (***), p < 0.01 (**), p < 0.05 (*) and p ≥ 0.05 (ns).

Kaplan-Meier survival analyses were performed to compare overall survival, listing-free survival, LVAD-free survival, and transplant-free survival between the treatment and comparison groups. The overall survival rate was significantly higher in the treatment group compared to the comparison group with a hazard ratio of 0.2 [0.07, 0.62] (p < 0.01) (Figure 5A). In contrast, listing-free survival, LVAD-free survival, and transplant-free survival did not show statistically significant differences between the two groups (Figure 5B, C & D).

## Discussion

The present study, which evaluates outcomes from SG in the largest cohort of advanced heart failure patients with comorbid obesity prior to LVAD implantation and a matched comparison group, demonstrates that SG results in enhanced survival, improved LVEF and NYHA functional class, and greater reductions in BMI compared to standard care. Additionally, the treatment group showed trends in positive cardiac remodeling, and reductions in non-GDMT heart failure medication usage. Notably, the comparison group, despite achieving an average 6.6% BMI reduction, demonstrated progression of disease, with no change in LVEF, worsening NYHA class, and an increase in adverse structural changes in cardiac morphology. This divergence suggests that the benefits observed in the SG group could be attributable to mechanisms beyond weight reduction.

Prior to this study, most evidence on the potential benefits of SG to treat obesity in advanced heart failure patients was derived from studies on patients living with an LVAD.^28^, ^29^, ^30^, ^31^ Retrospective studies have demonstrated preimplant obesity is associated with increased morbidity in LVAD implantation, with similar mortality regardless of the level of obesity.^32^, ^33^, ^34^, ^35^ Despite this, studies have demonstrated safety, along with successful listing and transplantation in patients with an LVAD and obesity undergoing SG.^36^, ^37^, ^38^ One study of 198 patients with LVAD and class II obesity with a history of bariatric surgery compared to non-surgical controls reported fewer heart failure events and increased probability of transplant.^39^ A pooled analysis evaluating 271 patients undergoing bariatric surgery (95.6% received SG) during or after LVAD placement reported 67.4% of patients proceed to listing and 32.5% achieving transplantation.^16^ Modern clinical guidelines reflect this evidence, with bariatric surgery receiving a Class IIB recommendation for management of obesity in advanced heart failure patients, whilst acknowledging optimal timing for SG, either pre- or post- LVAD has not been defined.^25^ New evidence from the present study, which had lower progressions to listing (20%) and transplantation (15%), suggests that SG may be performed prior to LVAD implantation with favorable outcomes, and may improve LVAD related outcomes due to decreased pre-implant obesity. Additionally, whilst our study population may have had less clinically advanced disease, improvements in heart failure outcomes may obviate the immediate need for advanced therapies.

Unlike earlier studies evaluating pre-LVAD patients with obesity that included various bariatric procedures, all patients in the present analysis underwent SG, showing improvements in BMI, LVEF and NYHA, consistent with previous results.^23^, ^24^, ^25^, ^26^, ^40^, ^41^ Notably, these results support previous findings of positive trends in cardiac remodeling, decreased use of adjunctive heart failure medications, and improvements independent of weight loss.^26^, ^42^, ^43^ In other studies of patients with a history of heart failure undergoing a bariatric procedure, surgery was associated with a 45% reduction in mortality, a 28% reduction in heart failure hospitalizations and a 19% reduction in major adverse cardiac events.^44^, ^45^ This is similar to the results from the present analysis, albeit in a population of patients with greater disease severity and provides further support for the conclusion that SG is associated with enhanced survival in advanced heart failure patients with obesity.

New pharmacological therapies, particularly GLP-1 agonists, have been investigated among patients with obesity and HFrEF as an alternative to surgical intervention. Five patients with T2DM in our comparison group were treated with a GLP-1 agonist and achieved significant weight loss. However, prospective randomized studies found that the GLP-1 agonist liraglutide was not associated with improvements in survival or LVEF and was associated with increased rates of re-hospitalization and adverse heart failure outcomes.^46^, ^47^, ^48^ This is in contrast to the promising trial results in the setting of heart failure with preserved ejection fraction (HFpEF), which demonstrated improved symptoms and exercise tolerance following treatment with semaglutide.^49^ This suggests that in the setting of advanced heart failure, the safety and efficacy of treatment with GLP-1 agonists is as yet unresolved and may not obviate the need for bariatric surgery in affected patients.^50^

While the present study provides valuable insights into the impact of SG on patients with advanced heart failure, several limitations should be acknowledged. The comparative rarity of SG in patients with advanced heart failure and obesity necessitated use of a retrospective study design. Such a study confers an increased risk of recall bias, selection bias and confounding. A comparison group was used to strengthen the study, and while drawn from the same hospital precinct, represented an overlapping but differing population of patients, who may not have been receiving specialist care through an advanced heart failure service. VO2 and comprehensive hemodynamic data were not systematically available in this cohort, though such measures would provide valuable insights in future studies. Although the present study represents the largest study conducted to date, the study sample is comparatively small, increasing the risk of type II errors. The exploratory nature of the analysis, combined with multiple comparisons, complicates the interpretation of findings, though significance calculations were adjusted to reduce the risk of type I errors. The relative strengths of this study include: examination of the largest cohort to date; use of a matched comparison group of patients with similar morbidity, receiving contemporaneous standard care; and a median follow-up period of 5 years, which enabled meaningful examination of outcomes.

In conclusion, SG is associated with significant improvements in cardiac function and survival in patients with HFrEF and obesity. Importantly, despite modest weight loss in the comparison group, there was evidence of disease progression, suggesting the degree of weight loss could play an important role, or additional mechanisms beyond weight loss could contribute to cardiac improvements. The results of this study suggest that SG is a safe and effective intervention for patients with HFrEF and obesity and may serve as a bridge to recovery or transplant candidacy.

## CRediT authorship contribution statement

Thomas Goubar contributed to study design, data acquisition, analysis, and drafting of the manuscript. Samuel Kim and David Cistulli assisted in data collection and analysis. Douglas Fenton-Lee and Peter S. Macdonald provided critical revisions of the manuscript for important intellectual content. R. Louise Rushworth contributed to the study's statistical analysis and revisions of the manuscript. Anne M. Keogh provided input on the study design and supervised the overall project. All authors reviewed, edited, and approved the final manuscript.

## Declaration of Interests

The authors declare that they have no known competing financial interests or personal relationships that could have appeared to influence the work reported in this paper.
